# Pollen *Streptomyces* Produce Antibiotic That Inhibits the Honey Bee Pathogen *Paenibacillus larvae*

**DOI:** 10.3389/fmicb.2021.632637

**Published:** 2021-02-04

**Authors:** Kirk J. Grubbs, Daniel S. May, Joseph A. Sardina, Renee K. Dermenjian, Thomas P. Wyche, Adrián A. Pinto-Tomás, Jon Clardy, Cameron R. Currie

**Affiliations:** ^1^Department of Bacteriology, University of Wisconsin-Madison, Madison, WI, United States; ^2^Department of Cellular and Molecular Pathology, University of Wisconsin-Madison, Madison, WI, United States; ^3^Laboratory of Genetics, University of Wisconsin-Madison, Madison, WI, United States; ^4^Department of Biological Chemistry and Molecular Pharmacology, Harvard Medical School, Boston, MA, United States

**Keywords:** pollen, *Apis mellifera*, piceamycin, *Streptomyces*, American foulbrood, *Paenibacillus larvae*, natural product

## Abstract

Humans use natural products to treat disease; similarly, some insects use natural products produced by Actinobacteria to combat infectious pathogens. Honey bees, *Apis mellifera*, are ecologically and economically important for their critical role as plant pollinators and are host to diverse and potentially virulent pathogens that threaten hive health. Here, we provide evidence that Actinobacteria that can suppress pathogenic microbes are associated with *A. mellifera*. We show through culture-dependent approaches that Actinobacteria in the genus *Streptomyces* are commonly isolated from foraging bees, and especially common in pollen stores. One strain, isolated from pollen stores, exhibited pronounced inhibitory activity against *Paenibacillus larvae*, the causative agent of American foulbrood. Bioassay-guided HPLC fractionation, followed by NMR and mass spectrometry, identified the known macrocyclic polyene lactam, piceamycin that was responsible for this activity. Further, we show that in its purified form, piceamycin has potent inhibitory activity toward *P. larvae*. Our results suggest that honey bees may use pollen-derived Actinobacteria and their associated small molecules to mediate colony health. Given the importance of honey bees to modern agriculture and their heightened susceptibility to disease, the discovery and development of antibiotic compounds from hives could serve as an important strategy in supporting disease management within apiaries.

## Introduction

Natural products play a critical role in human health, serving as drugs or drug leads that are used to treat diverse human afflictions, including cancer and hypertension (Newman and Cragg, 2020). Their role as antibiotics to treat infectious disease has had the largest impact on human health (Demain, 2009; Lewis, 2012). Most of the antibiotics used pharmaceutically are derived from Actinobacteria, especially soil dwelling members of the genus *Streptomyces*. Thousands of natural products have been discovered from Actinobacteria, and bioinformatic analysis of sequenced genomes has revealed that past efforts have overlooked many potentially useful compounds (Bachmann et al., 2014; Baltz, 2017). Despite their critical importance to human health, our understanding of the function of these secondary metabolites in their natural context is limited.

In addition to their use by humans, Actinobacteria and their natural products are known to be used by some insects to combat pathogens. One of the most well-studied systems is the complex and ancient symbiosis associated with fungus-growing ants. Many of these ants engage in an obligate mutualism with Actinobacteria in the genus *Pseudonocardia*, which produce antibiotics that help defend against parasitic infections of the mutualist fungus that the ants cultivate for food (Currie and Scott, 1999; Currie et al., 2002; Cafaro et al., 2011; Li et al., 2018). Similar to the symbiosis between fungus-growing ants and *Pseudonocardia*, digger wasps (*Philanthus* spp.; Kaltenpoth et al., 2005, 2006; Kroiss et al., 2010; Kai et al., 2018) and southern pine beetles (*Dendroctonus frontalis*; Scott et al., 2008) associate with antibiotic-producing *Streptomyces* in defensive mutualisms. Targeted isolations, culture-independent studies, and biochemical characterization of *Streptomyces* associated with solitary mud dauber wasps, termites, dung beetles, and other insects have suggested that such associations occur frequently (Poulsen et al., 2011; Visser et al., 2012; Kim et al., 2014; Um et al., 2016; Klassen et al., 2019; Song et al., 2019; Hwang et al., 2020). Indeed, a recent study of 2,561 insects spanning 15 orders and more than 10,000 *Streptomyces* isolates suggests that these types of defensive mutualisms are likely more widespread than currently recognized and may serve as a source for discovering novel natural products that could serve as antibiotic drug leads (Chevrette et al., 2019).

**Figure 1:**
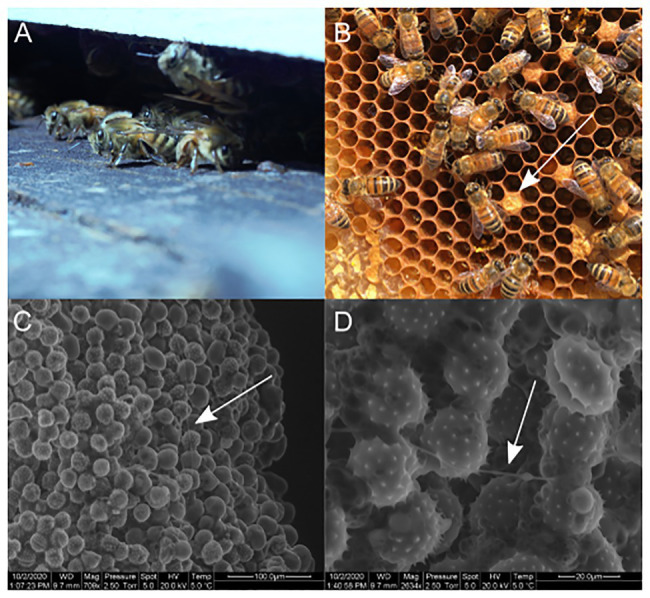
**(A)** *Apis mellifera*. **(B)** Pollen stores in comb structure of the hive. **(C)** SEM of pollen stores. **(D)** SEM of pollen stores with filamentous growth along pollen grains.

Honey bees (*Apis mellifera*) are social organisms that live in very dense colonies of closely related individuals, which makes honey bees more vulnerable to pathogens through increased pathogen transmission and host susceptibility (Schmid-Hempel, 1998; Evans and Schwarz, 2011; Figure 1A). A number of pathogens and parasites affect honey bees including viruses, bacteria, fungi, and mites (Bailey and Ball, 1991; Chen and Siede, 2007). *Paenibacillus larvae* (Firmicutes: Bacillales), the causative agent of American foulbrood, is one of the most widespread and destructive pathogens of honey bees. This honey bee specific pathogen is a spore forming Gram-positive bacteria that infects the larvae of honey bees through its highly infectious spores. The spores are consumed by larvae and germinate and grow vegetatively in the larval midgut leading to bacteremia and the formation of new spores. These spores are then spread throughout the hive and to other hives by contaminated worker bees, infecting new larvae as they are deposited in the hive environment (Genersch, 2010; Grady et al., 2016). To defend themselves and their hives from microbes, honey bees have developed a number of alternative immune and resistance strategies, including hygienic behavior (Rothenbuhler, 1964; Spivak and Reuter, 2001), use of antimicrobial materials in nest construction (Przybyłek and Karpiński, 2019), social fever (Starks et al., 2000), immune trait transference (Traniello et al., 2002; Sadd et al., 2005), and increased risk-taking behaviors by diseased individuals (Schmid-Hempel, 2005). Nevertheless, the presence of defensive symbionts might be expected within honey bee colonies. Indeed, recent interest in the honey bee gut and hive microbiota has identified the symbiotic gut bacteria *Snodgrassella alvi* and hive bacteria *Bombella apis* which are both capable of defending against parasites and opportunistic pathogens. In addition, other bee species have been shown to have associations with antibiotic-producing Actinobacteria including stingless bees in South and Central America and Asian honey bees in Thailand (Promnuan et al., 2009; Cambronero-Heinrichs et al., 2019; Menegatti et al., 2020).

In this study, we use Actinobacteria-specific culture methods and multi-gene phylogenetics to identify a diverse set of *Streptomyces* strains associated with honey bees and their hives. Growth inhibition assays were used to identify *Streptomyces* strains capable of inhibiting *P. larvae*, the causative agent of American foulbrood, and other insect and plant pathogens. Additionally, analytical chemistry and genome mining techniques were used to identify antibiotics capable of inhibiting pathogens of honey bees and their hives.

## Materials and Methods

### Isolation of Actinobacteria

We focused our sampling of honey bees and their different hive components from eight different hives. One hive in Wild Rose, WI, United States, two hives in Verona, WI, United States, three hives in Waukesha, WI, United States, and two hives in Mt. Horeb, WI, United States were sampled in 2007, 2008, 2008, and 2010, respectively. Honey, pollen stores, propolis, empty combs, bees in the hive, pupae, and newly eclosed bees emerging from brood cells were sampled from each hive. Additionally, four starter packages of bees, including workers and a queen, were obtained and sampled from Bee Charmer, Brooklyn, WI, United States and CA, United States (commercial bees). A swarm formed from a honey bee colony maintained in the Currie laboratory at the University of Wisconsin-Madison in 2008 was located and the swarming bees were sampled. Finally, foraging bees were found at flowers and fruit around Madison, WI, United States and sampled.

Enrichment isolation for Actinobacteria was conducted on single individuals for worker bees and pupae, and on a single cell for comb and pollen stores. For honey and propolis, we used 10 μl and 100 mg, respectively. Samples were homogenized in 500 μl of sterile diH_2_O by shaking in a beadbeater for 3 min without beads. For each sample type, 10 biological replicates were completed. Once homogenized, 100 μl of sample was plated in duplicate on chitin solid media (agar 20 g/L, unbleached chitin 4 g/L, K_2_HPO_4_ 0.77 g/L, MgSO_4_ × 7H_2_O 0.5 g/L, KH_2_PO_4_ 0.37 g/L, FeSO_4_ × 7H_2_O 0.01 g/L, MnCl_2_ × 4H_2_O 0.001 g/L, and ZnSO_4_ × 7H_2_O 0.001 g/L) with cyclohexamide (0.05 g/L) and nystatin (0.032 g/L) added to suppress the growth of fungus. Plates were allowed to dry, wrapped with parafilm, and then incubated at 30°C for a minimum of 4 weeks or until microbial growth covered the plate. Colonies exhibiting spores and/or a concentric ring growth pattern were enumerated, then subcultured onto chitin plates to obtain pure cultures, and subsequently grown on yeast malt extract agar medium (YMEA: yeast extract 4 g/L, malt extract 10 g/L, dextrose 4 g/L, and agar 15 g/L).

### Scanning Electron Microscopy

Environmental scanning electron microscopy (SEM) was used to visualize filamentous bacteria in pollen stores. Pollen samples were stored for 2 weeks at 4°C before imaging. Samples were mounted on SEM stubs with carbon bi-adhesive tabs, and images were taken at 2.5 torr, 5.0 spot size, and 5°C using a FEI QUANTA 200 eSEM.

### DNA Extraction

DNA was extracted from isolates *via* a CTAB Phenol/Chloroform protocol. Briefly, approximately 15 μl of bacterial tissue was scraped from cultures and homogenized *via* mortar and pestle in a 1.7 ml centrifuge tube containing 500 μl of a 2x CTAB solution. To the resulting slurry, 500 μl of a phenol:chloroform:isoamyl alcohol solution (25:24:1) was added. After vigorous mixing, the solution was centrifuged at max speed for 15 min and the resulting upper aqueous phase was removed and mixed with 500 μl of a chloroform:isoamyl alcohol (24:1) solution. The resulting solution was mixed and centrifuged at max speed for 15 min. The aqueous phase was then removed and combined with an equal volume of ice-cold isopropanol. Samples were incubated at −20°C overnight to allow DNA precipitation. Samples were then centrifuged at max speed for 30 min at 4°C. Resulting DNA was rinsed three times with 70% ethanol, air dried, resuspended in TE buffer, and stored at −20°C for future use.

### 16S Sequencing and Core-Genomic Phylogeny

Primers 27f and 1392r were used to obtain 16S sequences in PCR reactions with cycle parameters of 95°C for 2 min, 35 cycles of 95°C for 45 s, 55°C for 45 s, 72°C for 90 s, and 72°C for 5 min and hold at 4°C. Products were cleaned using ExoSapIT (USB) according to the manufacturer’s protocol. Sequencing reactions consisted of 2 μl of BigDye Terminator v. 3.1 mix (Applied Biosystems), 3 μl of dilution buffer (Applied Biosystems), 5–20 pmol of primer, and 0.2 μg of template DNA in a final reaction volume of 20 μl. Cycle conditions were 95°C for 3 min, then 35 cycles of 95°C for 20 s, 45°C for 30 s, and 60°C for 4 min, followed by 7 min at 72°C. Excess dye terminators were removed using CleanSeq magnetic beads (Agencourt Biosciences). Samples were then resuspended in 40 μl of ddH_2_O and sequenced at the University of Wisconsin-Madison Biotechnology Center using an Applied Biosystems 3730xl automated DNA sequencing instrument, using 50 cm capillary arrays and POP-7 polymer.

16S sequences were assembled using Bionumerics v6.5 (Applied Maths). Sequences were aligned in MEGAX (Kumar et al., 2018) using the MUSCLE algorithm. The alignment was inspected in MEGAX to ensure consistent reading frame, accurate gap placement, and reduction of sequence over hang at the ends. The model test module of MEGAX was used to ensure accurate choice of substitution model, and maximum likelihood phylogenies were inferred using 200 bootstrap replicates.

A multilocus phylogeny was created from the sequenced genomes of eight diverse bee-associated *Streptomyces* strains using methods described in (Chevrette et al., 2019). In brief, prodigal v2.6.0 (Hyatt et al., 2010) was used to call genes for each genome and HMMER v3.1b2 (Eddy, 2011) was used to search the genomes for 93 TIGRFAM amino acid sequences in the “core bacterial protein” set (GenProp0799). Protein families were aligned using MAFFT v7.245 (Katoh and Standley, 2013) and converted to codon alignments. RAxML v8.1.24 (Stamatakis, 2014) was used to generate phylogenetic trees for each codon alignment using the GTR gamma substitution model with 100 bootstraps. Finally, ASTRAL-II (Mirarab and Warnow, 2015) was used to generate the species phylogenetic tree from the individual codon aligned trees with 100 bootstraps. FigTree v1.4.3 was used to root the tree and display branch lengths proportional to the root. 16S sequences can be found on GenBank with accession numbers MW444694-MW444787.

### Growth Inhibition Bioassays

All isolates were assessed for growth inhibition against the honey bee specific pathogen American foulbrood (*P. larvae*), as well as an insect generalist pathogen *Beauveria bassiana* and a plant generalist pathogen *Fusarium oxysporum*. Actinobacteria isolates were inoculated in the center of 85 mm diameter YMEA plates and incubated at 30°C for 4 weeks. Fungi were then inoculated on the edge of the plate and incubated at 30°C until controls without bacterial isolates occupied half or more of the plate. Bacterial pathogens were grown in fresh liquid cultures and spread in the areas where the isolates had not grown. The area of fungal pathogens was measured using ImageJ (Schindelin et al., 2015) and compared to control fungal areas using an unpaired two-sample *t*-test. The minimum distance between isolate and pathogen was measured in bacterial pathogen assays and compared to zero using a single sample *t*-test.

### Analytical Chemistry Methods and Instrumentation

NMR experiments were performed in DMSO-d_6_ with a symmetrical NMR microtube (Shigemi, Inc.) on a Varian INOVA 600 MHz NMR. HPLC fractionation was performed on an Agilent 1100 Series HPLC system. LC/MS analysis was performed on an Agilent 1200 Series HPLC/6130 Series mass spectrometer. High resolution mass spectra were obtained on a Waters Micromass Q-Tof Ultima ESI-TOF mass spectrometer. Circular dichroism spectrum was obtained on an Aviv Biomedical Inc. (Lakewood, NJ, United States) Circular Dichroism Spectrometer, Model 410. Optical rotation measurements were obtained using a Jasco R-2000 digital polarimeter with a sodium lamp.

### Isolation and Identification of Piceamycin

*Streptomyces* sp. AmelAP-1 was isolated from pollen stores and maintained on YMEA at room temperature. Single colonies were used to inoculate 10 ml YME broth (4 g yeast extract, 10 g malt extract, and 4 g dextrose per L) seed cultures, which were incubated at 25°C with shaking at 250 rpm for 48 h. This seed was then used to inoculate 500 ml cultures of YME broth, also incubated at 25°C, 250 rpm for 48 h. Extraction and fractionation procedures were performed in the dark due to photosensitivity of piceamycin. Bacterial cultures were centrifuged and the supernatant extracted twice with an equal volume of ethyl acetate. The organic extract was dried over sodium sulfate, and then concentrated *in vacuo*. The extract was then resuspended in a minimum volume of methanol, and adsorbed onto celite. This mixture was dried, loaded onto a 2 g C_18_ Sep-Pak SPE cartridge, and fractionated by eluting with a step gradient of methanol/water. The 80% methanol fraction was most active, as deduced by spotting fractions onto lawns of *Bacillus subtilis* on LB agar and assessing zones of inhibition. This fraction was subsequently purified by reverse-phase HPLC with UV detection using a semi-preparative Discovery HS-C_18_ column (Supelco, 25 cm × 10 mm, 10 μm particle size) with an acetonitrile:water gradient at 3 ml/min: 0–15 min, 55% MeCN; 15–17 min, 55–100% MeCN. Piceamycin eluted at ~15 min. The overall yield of piceamycin is approximately 0.6 mg/L. For acetylation, piceamycin was dissolved in pyridine, and a stoichiometric amount of acetic anhydride was added. The reaction was stirred at room temperature for 1 h, evaporated to dryness, and redissolved in methanol for LC/MS analysis.

### Genome Sequencing and Biosynthetic Gene Cluster Identification

DNA was isolated as described above and prepared for Illumina MiSeq 2 × 300 bp paired-end sequencing by the University of Wisconsin-Madison Biotechnology Center. The resulting reads were corrected with MUSKET v1.1, and paired-ends were merged with FLASH v1.2.7 and finally assembled with SPAdes v3.11.0. The assembly (GenBank assembly accession: GCA_009865135.1) was submitted to AntiSMASH v5.0 for the identification of biosynthetic gene clusters. A putative piceamycin polyketide synthase was identified based on high similarity to the published bombyxamycin/piceamycin polyketide synthase. The two biosynthetic gene clusters were directly compared by BLAST and MultiGeneBlast v1.1.14 (Medema et al., 2013).

### Antibacterial Assays of Piceamycin

All bioassays were performed in triplicate. Overnight cultures of all strains assayed were diluted to ~10^6^ cells/ml in LB and transferred to sterile 96-well plates (200 μl per well). A serial dilution of piceamycin dissolved in DMSO or only DMSO, as a negative control, was added to the cultures, and the plates were incubated without shaking at 30°C (for *Paenibacillus* and *Bacillus* strains) or 37°C (for *Escherichia coli*). OD_600_ measurements were performed on a Molecular Devices SpectraMax® M5 plate reader after 15 h.

## Results

*Apis mellifera* pupae, newly eclosed bees, adult bees found in hives, adult bees found foraging (found at flowers or fruit), swarming bees, commercial bees, empty combs, propolis, honey, and pollen stores were all sampled for the presence of Actinobacteria. Enrichment isolation for Actinobacteria resulted in at least one isolate from all sampled material (Table 1; Supplemental Table 2). Fewer actinobacterial colony forming units (CFUs) were isolated from the bee brood or hive environment such as pupae, newly eclosed workers, honey, empty combs, or propolis. Actinobacterial CFUs were more common from older adult bees such as hive bees, foraging bees, swarming bees, and commercial bees. Pollen stores, however, consistently showed the greatest number of actinobacterial CFUs (Figure 1B; Table 1). Scanning electron microscopic (SEM) examination of honey bee adults did not identify Actinobacteria; however, SEM of pollen stores in hives revealed growth consistent with Actinobacteria morphology (Figures 1C,D).

**Table 1:** Average colony forming units (CFU) count from bee and bee environment sources.

Source	Average CFU (SEM)
Pupae	1.1 (0.97)
Newly eclosed workers	0.33 (0.2)
Adult hive bees	5.6 (1.3)
Adult foraging bees	9.4 (1.4)
Swarming bees	13.8 (7.3)
Commercial bees	14 (3.4)
Honey	0.05 (0.04)
Empty comb	0.91 (0.56)
Propolis	0.95 (0.68)
Pollen stores	25.4 (3.0)

Ten biological replicates were collected from each source and SEM is shown in parentheses.

**Figure 2:**
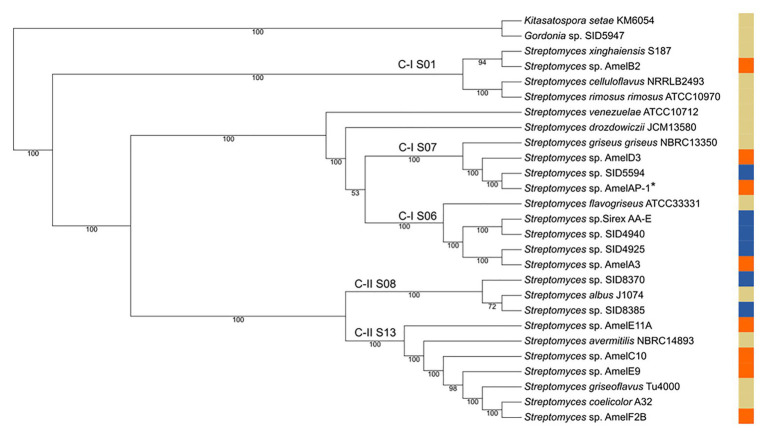
Core genome tree of select honey bee associated *Streptomyces* (orange), reference *Streptomyces* strains (tan), and other insect associated *Streptomyces* strains (blue). Clades are labeled in reference to Chevrette et al. (2019) with C-I S06, C-I S07, and C-II S08 representing clades enriched for insect-associated *Streptomyces*. AmelAP-1 is marked by a * in the tree.

*Streptomyces* isolates from bees and hive material were selected for genome sequencing based on a preliminary 16S rDNA analysis. Eight strains were selected to represent the diversity of *Streptomyces* strains observed in the 16S rDNA analysis. The strains were sequenced and assembled, and a phylogenomic tree of the strains was created based on 93 core genes. Both the 16S analysis and the core genome tree demonstrate a diversity of *Streptomyces* can be isolated from honey bees and their hives (Figure 2; Supplementary Figure 1). Three of the strains in the core genome tree are in clades that are enriched with insect-associated symbiotic *Streptomyces* spp., AmelAP-1, AmelD3, and AmelA3. Based on the phylogeny in Chevrette et al. (2019), *Streptomyces* spp. AmelAP-1 and AmelD3 are in Clade I S07 and are closely related to several *Streptomyces* strains isolated from solitary mud dauber wasps. *Streptomyces* sp. AmelA3 is in Clade I S06 and is closely related to *Streptomyces* strains isolated from mountain pine beetles and fungus-growing termites. Other strains are located widely throughout the genus *Streptomyces*.

All isolated *Streptomyces* were screened in pairwise assays against *P. larvae* the causative agent of American foulbrood, *Beauveria bassiana* an entomopathogenic fungus, and *Fusarium oxysporum* a common plant pathogen. Each isolate demonstrated inhibition against at least one pathogen with several inhibiting only the bacterial pathogens or inhibiting only the fungal pathogen (Supplemental Table 2). *Streptomyces* sp. AmelAP-1, isolated from pollen stores, was found to inhibit all three pathogens and was particularly active against *P. larvae*. Additionally, *Streptomyces* AmelAP-1 was shown to be closely related to other insect associated *Streptomyces* strains; therefore, it was chosen for further analysis.

AmelAP-1 was grown in Yeast Malt-extract media in liquid culture and the supernatant was extracted with ethyl acetate and dried *in vacuo* to yield a crude extract. The extract was confirmed to be active against *P. larvae* and bioactivity guided HPLC fractionation revealed a pure compound (1) with strong inhibition against *P. larvae* ([*α*]_D_^28^ 74.4 (c = 0.34, CH_3_OH)). HRESIMS analysis of this compound (*m/z* 432.2163) indicated a molecular formula of C_27_H_29_NO_4_ (calcd. *m/z* for [M + H]^+^, 432.2169). The fourteen degrees of unsaturation indicated by this formula, coupled with the absorption spectrum (*λ*_max_ = 410 nm) and extreme photosensitivity of the compound suggested a polyene moiety. This was confirmed by NMR analysis using one- and two-dimensional NMR techniques including ^1^H, gCOSY, gHSQC, gHMBC, and ROESY.

**Figure 3:**
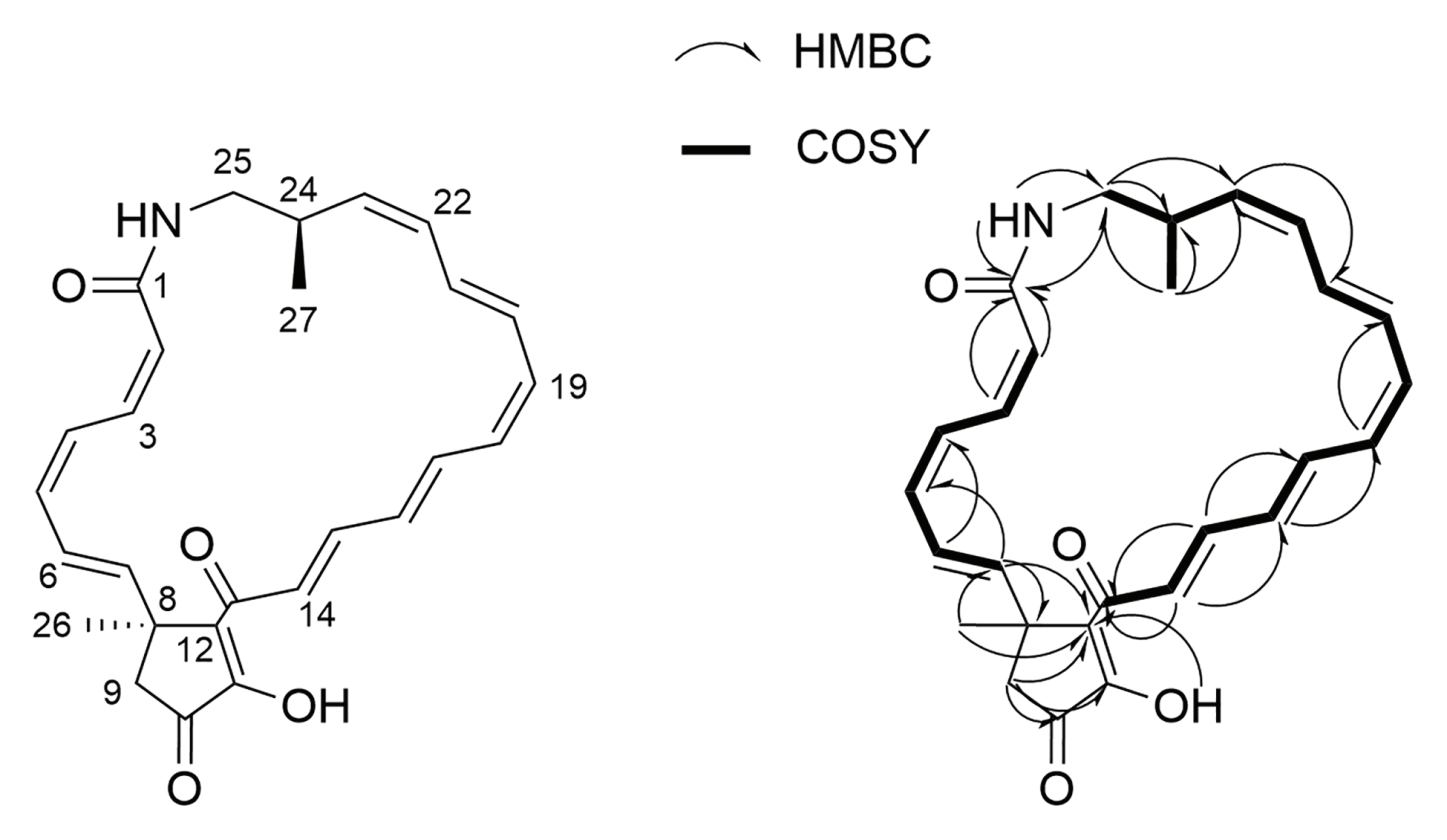
Key COSY and HMBC correlations used for the confirmation of piceamycin.

Two distinct polyene-containing spin systems were detected by ^1^H and gCOSY experiments (Figure 3). Configurations of all double bonds in these units were determined by analysis of proton coupling constants and ROESY correlations. HSQC and long-range HMBC correlations established the presence of a cyclopentenone moiety linking these two polyene units at one end, and an amide bond closing the 23-membered lactam ring at the other (Figure 3). To confirm assignment of the hydroxy group, the compound was acetylated with acetic anhydride in pyridine. Subsequent mass spectral analysis revealed a mass of *m/z* 473.2 [M + H]^+^, corresponding to acetylation at the only hydroxy group.

The planar structure of 1 was identical to the previously reported piceamycin (Schulz et al., 2009); however, there were significant differences in the ^13^C chemical shifts of the cyclopentenone moiety, suggesting that 1 could be an isomer of piceamycin (Table 2). A ROESY correlation observed between the two methyl groups combined with minimum energy modeling of the possible stereoisomers indicated that the methyl groups are likely to be within range for coherence transfer in the 24R, 8S configuration. This is the same configuration as reported by Shin et al. (2020), suggesting that 1 is piceamycin; however, this does not explain the deviation of the cyclopentenone ^13^C chemical shifts. Further NMR experiments were hindered by the photosensitivity of the compound, but four peaks with identical *m/z* values were detected by HR-LCMS in the extract of AmelAP-1, suggesting that several conformational isomers may be present in the extract with one at much higher abundance than the others.

**Table 2:** ^1^H and ^13^C assignments of piceamycin (1) in d_6_-DMSO.

#	δ_C_	Type	δ_H_	mult (*J*, Hz)
1	163.9	C		
2	121.2	CH	5.06	d (12.9)
3	132.5	CH	6.26	dd (12.7)
4	122.5	CH	7.07	dd (11.4)
5	134.4	CH	6.13	dd (11.4)
6	119.9	CH	5.99	dd (11.4, 14.7)
7	147.1	CH	5.92	d (14.7)
8	40.8	C		
9	49.4	CH_2_	2.04	d (18.9)
			2.06	d (18.9)
10	213.3	C		
11	171.0	C		
11-OH			6.74	br s
12	127.6	C		
13	188.8	C		
14	133.4	CH	8.01	d (15.4)
15	136.1	CH	6.81	dd (15.4, 11.4)
16	130.8	CH	6.43	dd (14.2, 11.4)
17	133.2	CH	6.89	dd (14.2, 10.4)
18	128.0	CH	6.20	m (<11)^a^
19	130.7	CH	6.22	m (<11)^a^
20	127.3	CH	6.64	dd (15.2, 8.7)
21	130.4	CH	6.53	dd (15.0, 10.4)
22	129.1	CH	6.06	dd (10.4)
23	135.2	CH	5.07	m^a^
24	33.2	CH	2.67	m^a^
25	43.6	CH_2_	2.64	m^a^
			3.35	m^a^
26	29.2	CH_3_	1.53	s
27	17.6	CH_3_	0.95	d (6.3)
NH			7.40	d (9.9)

a Overlapped peaks; coupling constant unknown.

**Figure 4:**
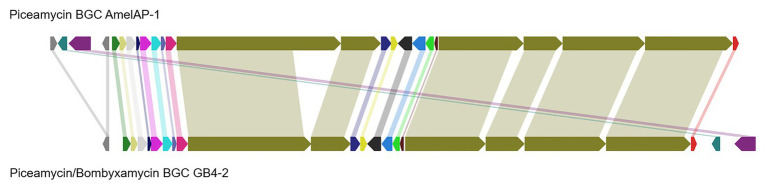
Comparison of the identified polyketide synthase (PKS) biosynthetic gene cluster (BGC) in AmelAP-1 to the published BGC for bombyxamycin and piceamycin. Shaded bars between BGCs identify homologous genes between the two BGCs.

To provide further evidence that 1 is piceamycin, we examined the biosynthetic gene clusters present in the genome of *Streptomyces* sp. AmelAP-1 using AntiSMASH 5.0 (Blin et al., 2019). A polyketide synthase (PKS) biosynthetic gene cluster (BGC) with homology and synteny to that of the reported bombyxamycin and piceamycin biosynthetic gene cluster was identified (Shin et al., 2020). A comparison of the two BGCs was performed using BLASTP and MultiGeneBlast. Each gene in the bombyxamycin and piceamycin biosynthetic gene cluster contained a homologous gene in the PKS identified in the genome of AmelAP-1 (Figure 4; Supplemental Table 1). The PKS in AmelAP-1 contains six PKS genes composed of 12 modules. This deviates from the published piceamycin BGC that has six PKS genes composed of 11 modules. The biosynthetic gene cluster in AmelAP-1 also contains a duplicated type II thioesterase gene, which may be responsible for skipping or off-loading the growing polyketide chain from one of the extra modules containing a putatively inactive domain (Heathcote et al., 2001; Wu et al., 2018). The homology and synteny to the published bombyxamycin and piceamycin biosynthetic gene cluster suggests that the identified PKS is putatively responsible for the production of piceamycin in AmelAP-1.

**Figure 5:**
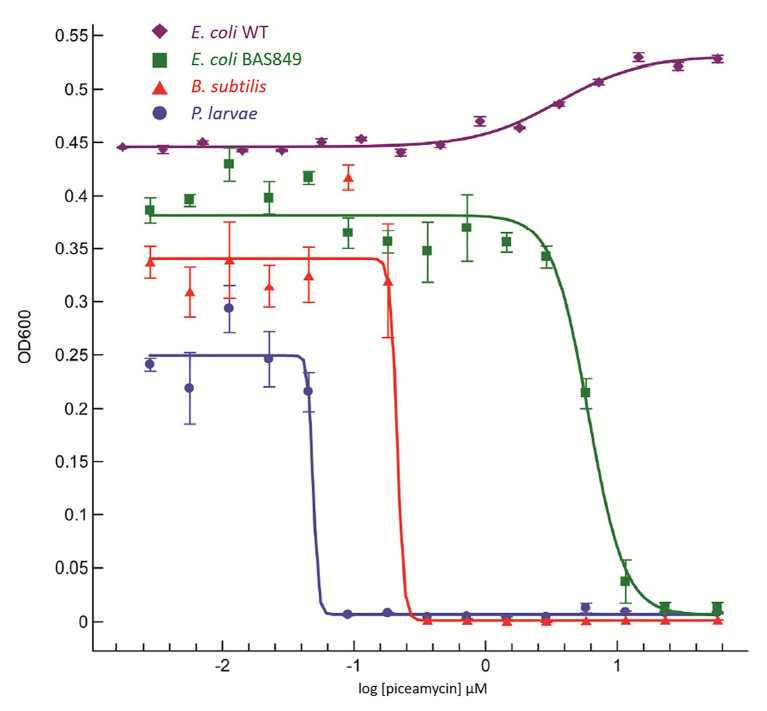
Dose response curve using piceamycin against *Paenibacillus larvae*, *Bacillus subtilis*, and two strains of *Escherichia coli*.

A growth inhibition bioassay using purified piceamycin at decreasing concentrations was used to determine the activity and specificity of the compound toward *P. larvae* (Figure 5). Piceamycin was found to have high inhibition of *P. larvae*, with a minimum inhibitory concentration (MIC) of 48 nM. *Bacillus subtilis* and *E. coli* BAS849 were less sensitive with MICs of 213 nM and 6 μM, respectively. Growth of the wild-type *E. coli* K12 was not inhibited at the concentrations tested.

## Discussion

Here, we identified a known antibiotic produced by a *Streptomyces* isolated from the pollen stores of honey bees. The compound was confirmed based on NMR and LC-MS data as well as the identification of a PKS gene cluster with homology and synteny to the reported piceamycin biosynthetic gene cluster. Piceamycin is a macrocyclic polyene lactam, which has been recently reported in association with other insects (Shin et al., 2020). We reveal that piceamycin has high inhibitory activity, with a MIC of 48 nM, against *P. larvae*, a common and destructive Gram-positive bacterial pathogen of honey bees. Interestingly, this compound had significantly less inhibitory activity toward another closely related Gram-positive bacteria, *B. subtilis*, with a MIC of 213 nM. Additionally, a mutant *E. coli* that is sensitive to antibiotics due to a compromised outer membrane was only slightly inhibited by piceamycin, while wildtype *E. coli* was not inhibited at all. The apparent specificity of piceamycin toward *P. larvae* suggests that this small molecule could help mediate pathogen dynamics within honey bee colonies.

Diverse *Streptomyces* strains were isolated from adult bees in managed hives, purchased adult bees, swarming bees, and various parts of bee hives. Interestingly, the most numerous isolations consistently came from pollen stores, and filamentous, Actinobacteria-like, bacteria were observed on pollen stores using scanning electron microscopy. Similar findings were described in a recent publication describing the tripartite symbiosis of *Streptomyces*, honey bees (*Apis mellifera*), and strawberry plants (Kim et al., 2019). Other publications have also recently reported *Streptomyces* associated with various bee species and it is possible that many of these bee species are gathering beneficial *Streptomyces* strains through the pollen collected from plants. Many of these *Streptomyces* strains have the ability to inhibit both bee pathogens and plant pathogens, as was demonstrated by Kim et al. (2019), as well as by the inhibition of both *P. larvae* and *Fusarium oxysporum* by AmelAP-1 in this study. Additionally, several other strains isolated in our study were capable of inhibiting either *P. larvae* or other potential bee or bee hive pathogens; however, the small molecules responsible for the inhibition have not yet been identified. While there is no consistent phylogenetic signal of a single vertically transferred symbiont, it is possible that pollinators and their plants are using a diverse suite of protective bacteria that produce compounds capable of inhibiting both plant and pollinator pathogens.

The eusocial nature of honey bees leads to increased susceptibility to disease. The living together of closely related individuals in high density increases pathogen transmission and virulence within hives. Interestingly, despite this prediction, the genome of *A. mellifera* contains only a third the number of immune related genes than does the *Drosophila* genome (Evans et al., 2006). In response, honey bees have developed a number of alternative immune strategies, which may include the use of antibiotic-producing bacteria. The apparent use of natural products derived from bacteria by honey bees parallels the importance of natural products for treating pathogens by humans. Given the dramatic decrease in the discovery of novel antibiotics, this work and others suggest that *A. mellifera* and other bees may represent a valuable source for discovering novel antibiotics. Indeed, in the last several years studies have identified both Actinobacteria and Proteobacteria associated with the hive environment that can inhibit both bacterial and fungal pathogens of bees (Cambronero-Heinrichs et al., 2019; Menegatti et al., 2020; Miller et al., 2020; Smith and Newton, 2020). Additionally, the specificity of piceamycin makes it a prime candidate for development into a biological hive treatment to protect against American foulbrood. Given the apparent increase in pathogen pressure currently experienced by managed hives and our reliance on honey bees as important pollinators for a number of agriculture crops, piceamycin, and other compounds identified using similar approaches could have important implications for human agriculture and the apiculture industry.

## Data Availability Statement

The datasets presented in this study can be found in online repositories. The names of the repository/repositories and accession number(s) can be found in the article/Supplementary Material.

## Author Contributions

The collection, isolation, and screening of bee associated Streptomyces were performed by KG and AP-T. Scanning electron microscopy was performed by JS. 16S phylogenic analysis was performed by KG and DM and the core-genome phylogenic analysis was performed by DM. Isolation and analytical analysis of piceamycin were performed by TW and RD. Identification and analysis of the biosynthetic gene cluster that encodes the production of piceamycin in AmelAP-1 were performed by DM. The manuscript was written by DM and KG. Funding was acquired by KG, JC, and CC. All authors contributed to the article and approved the submitted version.

### Conflict of Interest

The authors declare that the research was conducted in the absence of any commercial or financial relationships that could be construed as a potential conflict of interest.
