# Supplementary material for: Pollen Streptomyces Produce Antibiotic That Inhibits the Honey Bee Pathogen Paenibacillus larvae
Source: Front Microbiol. 2021 Feb 4;12:632637. doi: 10.3389/fmicb.2021.632637 (PMC7889971; doi:10.3389/fmicb.2021.632637)
Supplement: Supplementary file 1 [file Table_1.pdf]

Supplemental Table 1: tBLASTn analysis comparing the AntiSMASH output biosynthetic gene cluster from AmelAP-1 to the published biosynthetic gene cluster for bombyxamycin and piceamycin from Strain GB4-2.

AmelAP-1 Piceamycin BGC	GB4-2 Bombyxamycin/ Piceamycin BGC	BLASTP ID %	Coverage %
ctg1_2969	BomR9	55.17	88
ctg1_2968	BomR5	55.4	52
ctg1_2967	BomR4	59	96
ctg1_2966	-	-	-
ctg_12965	QBL56203.1	75.3	100
ctg1_2964	BomR9	72.11	98
ctg1_2963	BomM	74.16	94
ctg1_2962	BomL	66	91
ctg1_2961	BomK	86	100
ctg1_2960	QBL56196.1	85.9	100
ctg1_2959	BomJ	77.7	96
ctg1_2958	BomI	72.66	99
ctg1_2957	BomH	72.7	86
ctg1_2956	BomG	72.36	87
ctg1_2955	BomP6	68.2	100
ctg1_2954	BomP5	62.3	98
ctg1_2953	BomF	71.2	91
ctg1_2952	BomR8	80.83	94
ctg1_2951	QBL56188.1	59.6	95
ctg1_2950	BomE	73.8	99
ctg1_2949	QBL56186.1	75.7	60
ctg1_2948	BomD	78.2	100
ctg1_2947	BomP4	61.8	96
ctg1_2946	BomP3	52.9	92
ctg1_2945	BomP2	61	100
ctg1_2944	BomP1	63	100
ctg1_2943	BomC	87	98
ctg1_2942	-	-	-
ctg1_2941	-	-	-
ctg1_2940	-	-	-
ctg1_2939	-	-	-
