# Supplementary material for: Pollen Streptomyces Produce Antibiotic That Inhibits the Honey Bee Pathogen Paenibacillus larvae
Source: Front Microbiol. 2021 Feb 4;12:632637. doi: 10.3389/fmicb.2021.632637 (PMC7889971; doi:10.3389/fmicb.2021.632637)
Supplement: Supplementary file 2 [file Table_2.pdf]

Supplemental Table 2: Strains isolated from honey bees and the hive environment. Organism determination was determined by 16S analysis. Inhibition of the fungal pathogens is represented by the ratio of the average surface area of the inhibited fungus over the average area of the control fungus. Inhibition of the bacterial pathogen is represented by the zone of inhibition in mm. P-values from unapired two sample (fungal pathogens) and single sample (bacterial pathogen) t-tests of the inhibition of the pathogens are given in parentheses. Red values indicate significant inhibition, black values indicate no signficance and blue values indicate significant growth.

Strain	Organism	Isolation Source	Inhibition *F oxysporum*	Inhibition *B bassiana*	Inhibition *P larvae*	Strain	Organism	Isolation Source	Inhibition *F oxysporum*	Inhibition *B bassiana*	Inhibition *P larvae*
AmelKG-A1	Streptomyces sp.	Newly Eclosed Worker				AmelKG-E8	Streptomyces sp.	Pollen Stores			
AmelKG-A10	Streptomyces sp.	Empty Comb				AmelKG-E9	Streptomyces sp.	Commercial Bee	0.41 (0.0308)	0.75 (0.0012)	0mm (0.5)
AmelKG-A11	Actinomadura sp.	Commercial Bee				AmelKG-F1	Streptomyces sp.	Hive Bee			
AmelKG-A12	Streptomyces sp.	Hive Bee				AmelKG-F10	Streptomyces sp.	Commercial Bee			
AmelKG-A2	Streptomyces sp.	Hive Bee				AmelKG-F11	Streptomyces sp.	Newly Eclosed Worker			
AmelKG-A3	Streptomyces sp.	Pollen Stores				AmelKG-F2	Streptomyces sp.	Pupae	0.34 (<0.0001)	0.36 (<0.0001)	0mm (0.5)
AmelKG-A4	Streptomyces sp.	Empty Comb				AmelKG-F3	Streptomyces sp.	Hive Bee			
AmelKG-A5	Streptomyces sp.	Hive Bee				AmelKG-F4	Streptomyces sp.	Hive Bee	0.33 (<0.0001)	0.35 (<0.0001)	0.7mm (0.1955)
AmelKG-A6	Streptomyces sp.	Pollen Stores				AmelKG-F5	Micromonospora sp.	Commercial Bee			
AmelKG-A7	Streptomyces sp.	Empty Comb				AmelKG-F6	Streptomyces sp.	Commercial Bee			
AmelKG-A8	Streptomyces sp.	Pollen Stores	0.37 (0.0032)	0.71 (0.0005)	4.1mm (0.1955)	AmelKG-F7	Streptomyces sp.	Commercial Bee			
AmelKG-A9	Streptomyces sp.	Pollen Stores				AmelKG-F8	Streptomyces sp.	Pollen Stores			
AmelAP-1	Streptomyces sp.	Pollen Stores	0.47 (0.0152)	0.62 (0.0015)	7.5mm (0.0352)	AmelKG-F9	Streptomyces sp.	Pollen Stores			
AmelKG-B1	Actinomadura sp.	Commercial Bee				AmelKG-G1	Streptomyces sp.	Pollen Stores			
AmelKG-B2	Streptomyces sp.	Hive Bee				AmelKG-G10	Micromonospora sp.	Newly Eclosed Worker			
AmelKG-B3	Saccharopolyspora sp.	Empty Comb				AmelKG-G11	Micromonospora sp.	Pollen Stores	1.0 (0.5224)	0.57 (0.0002)	11.4mm (0.0076)
AmelKG-B4	Streptomyces sp.	Foraging Bee				AmelKG-G12	Streptomyces sp.	Pollen Stores	0.18 (0.0072)	0.97 (0.429)	0mm (0.5)
AmelKG-B5	Streptomyces sp.	Hive Bee				AmelKG-G5	Micromonospora sp.	Commercial Bee			
AmelKG-B6	Streptomyces sp.	Pollen Stores	1.0 (0.1378)	1.24 (0.5772)	14.8mm (0.0015)	AmelKG-G6	Micromonospora sp.	Newly Eclosed Worker			
AmelKG-B7	Streptomyces sp.	Pollen Stores				AmelKG-G7	Micromonospora sp.	Commercial Bee			
AmelKG-B8	Streptomyces sp.	Foraging Bee				AmelKG-G8	Micromonospora sp.	Commercial Bee			
AmelKG-C1	Actinomadura sp.	Commercial Bee	0.91 (0.0021)	0.81 (0.0095)	15.1mm (0.0002)	AmelKG-G9	Micromonospora sp.	Commercial Bee			
AmelKG-C10	Streptomyces sp.	Pollen Stores	0.29 (<0.0001)	0.40 (<0.0001)	23.1mm (<0.0001)	AmelKG-H5	Streptomyces sp.	Swarming Bee			
AmelKG-C11	Streptomyces sp.	Pollen Stores				AmelKG-H6	Streptomyces sp.	Commercial Bee			
AmelKG-C12	Streptomyces sp.	Foraging Bee				AmelKG-10	Streptomyces sp.	Honey Bee			
AmelKG-C9	Streptomyces sp.	Pollen Stores	0.01 (<0.0001)	0.16 (<0.0001)	0mm (0.5)	AmelKG-11	Streptomyces sp.	Honey Bee			
AmelKG-D1	Streptomyces sp.	Hive Bee				AmelKG-12	Streptomyces sp.	Honey Bee			
AmelKG-D10	Streptomyces sp.	Commercial Bee				AmelKG-14	Streptomyces sp.	Honey Bee			
AmelKG-D11	Streptomyces sp.	Pollen Stores				AmelKG-15	Saccharopolyspora sp.	Honey Bee			
AmelKG-D12	Streptomyces sp.	Foraging Bee				AmelKG-17	Streptomyces sp.	Honey Bee			
AmelKG-D2	Streptomyces sp.	Pollen Stores				AmelKG-18	Streptomyces sp.	Honey Bee			
AmelKG-D3	Streptomyces sp.	Commercial Bee				AmelKG-19	Streptomyces sp.	Honey Bee	0.49 (0.0169)	0.60 (<0.0001)	0mm (0.5)
AmelKG-D4	Streptomyces sp.	Commercial Bee				AmelKG-20	Streptomyces sp.	Honey Bee	0.11 (0.0021)	0.92 (0.2025)	5.3mm (0.1089)
AmelKG-D5	Streptomyces sp.	Hive Bee				AmelKG-21	Streptomyces sp.	Honey Bee			
AmelKG-D7	Streptomyces sp.	Hive Bee				AmelKG-2	Streptomyces sp.	Honey Bee			
AmelKG-D8	Streptomyces sp.	Commercial Bee				AmelKG-22	Streptomyces sp.	Honey Bee			
AmelKG-D9	Streptomyces sp.	Commercial Bee	0.62 (0.104)	0.70 (0.0075)	4.7mm (0.1955)	AmelKG-3	Streptomyces sp.	Honey Bee	0.64 (<0.0001)	0.78 (0.0624)	12.6mm (0.0016)
AmelKG-E1	Saccharopolyspora sp.	Hive Bee	1.16 (0.0004)	1.11 (0.5602)	28.1mm (<0.0001)	AmelKG-4	Streptomyces sp.	Honey Bee			
AmelKG-E10	Streptomyces sp.	Commercial Bee				AmelKG-5	Streptomyces sp.	Honey Bee			
AmelKG-E11	Streptomyces sp.	Hive Bee	0.96 (0.068)	1.35 (0.003)	14.3mm (0.0063)	AmelKG-6	Streptomyces sp.	Honey Bee			
AmelKG-E12	Streptomyces sp.	Hive Bee	0.87 (0.1572)	0.48 (<0.0001)	7.2mm (0.1955)	AmelKG-7	Streptomyces sp.	Honey Bee			
AmelKG-E2	Streptomyces sp.	Pollen Stores				AmelKG-8	Streptomyces sp.	Honey Bee			
AmelKG-E3	Streptomyces sp.	Hive Bee				AmelKG-9	Streptomyces sp.	Honey Bee			
AmelKG-E4	Streptomyces sp.	Hive Bee				AmelKG-C2	Streptomyces sp.	Commercial Bee			
AmelKG-E5	Streptomyces sp.	Swarming Bee	0.88 (0.0003)	1.08 (0.5902)	16.9mm (0.0002)	AmelKG-C3	Streptomyces sp.	Commercial Bee			
AmelKG-E6	Streptomyces sp.	Foraging Bee	1.0 (0.7403)	0.80 (0.0071)	15.9mm (0.0084)	AmelKG-C91	Streptomyces sp.	Pollen Stores			
AmelKG-E7	Actinomadura sp.	Pollen Stores				AmelKG-F91	Streptomyces sp.	Pollen Stores			
