# Supplementary material for: Pollen Streptomyces Produce Antibiotic That Inhibits the Honey Bee Pathogen Paenibacillus larvae
Source: Front Microbiol. 2021 Feb 4;12:632637. doi: 10.3389/fmicb.2021.632637 (PMC7889971; doi:10.3389/fmicb.2021.632637)
Supplement: Supplementary file 3 [file Image_1.pdf]

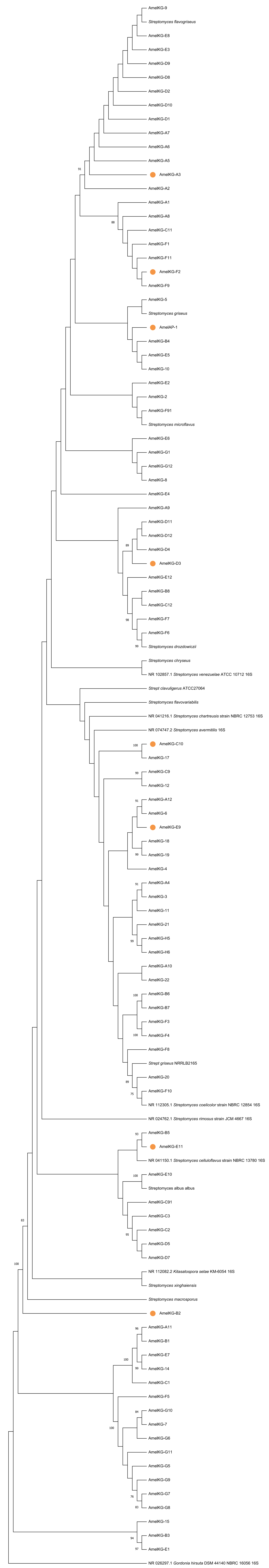

Supplemental Figure 1: Maximum Likelihood phylogeny of 16S sequences from honey bee associated *Streptomyces* and various reference *Streptomyces* strains. The tree was made with 200 bootstrap replicates and bootstrap values greater than 75% are shown on the tree. Orange circles denote strains that were selected for further sequencing and study.
